# Speaking the Same Language – The Development of a Glossary of Terms for Social Prescribing in Wales

**DOI:** 10.5334/ijic.8591

**Published:** 2024-07-01

**Authors:** Simon Newstead, Amrita Jesurasa, Bethan Jenkins, Amber Lavans, Alan Woodall, Carolyn Wallace

**Affiliations:** 1 Faculty of Life Sciences and Education, University of South Wales, UK; 2 Wales School for Social Prescribing Research (WSSPR), UK; 3 Public Health Wales, UK; 4 Powys Teaching Hospital Board, UK

**Keywords:** social prescribing, community connection, link worker, glossary of terms, scoping review, group concept mapping

## Abstract

**Introduction:**

Social prescribing can facilitate the integration of health, social care and community support but has a diverse and confusing terminology that impairs cross-sectoral communication and creates barriers to engagement.

**Methods:**

To address this issue a mixed-methods approach that incorporated a scoping review, a group concept mapping study and consultation was employed to identify and classify the terminology associated with social prescribing. The findings were then used to inform the development of a glossary of terms for social prescribing.

**Results:**

Many terms are used interchangeably to describe the same specific aspects of social prescribing. Much of the terminology originates from the health and social care literature of England.

**Discussion:**

The terminology used in the academic literature may not accurately reflect the terminology used by the social prescribing workforce. The innovative and interactive glossary of terms identifies the terminology associated with social prescribing and provides additional contextual information. The process of developing the dual language glossary presented several considerations and challenges.

**Conclusion:**

The glossary of terms will facilitate cross-sector communication and reduce barriers to engagement with social prescribing. It takes an important first step to help clarify and standardise the language associated with social prescribing, for professionals and members of the public alike.

## Introduction

Social prescribing provides a means to facilitate the integration of healthcare, social care and community support, allowing individuals to take an active role in improving their health and wellbeing [1,2,3]. Primary care workers can become overwhelmed by the psycho-social issues that their patients present with [4,5]. In line with the Social Services and Wellbeing (Wales) Act [6] and the Wellbeing and Future Generations (Wales) Act [7], Wales has developed a cross-sectional model of social prescribing that moves away from the medical model of care and is integrated with existing community and statutory services [8,9]. This model focuses on holistic and person-centred methods to improve wellbeing [10] with consideration of the biomedical, psychological and social dimensions of health and wellbeing [11,12].

The ‘prescribing’ element of the term social prescribing might imply that the individual is ‘told what they need to do’. However, within Wales, social prescribing describes a pathway which uses a person-centred approach to empower individuals to better manage their health and wellbeing through engagement with different community-based initiatives and activities [13,14,15,16,17]. One key aspect of social prescribing is the ‘what matters conversation’, during which the social prescribing practitioner supports the individual to identify their needs and what is important to them [18]. Following this exchange the social prescribing practitioner and the individual co-produce goals and an action plan outlining how these goals will be met [18]. By engaging with this process, the individual gains increased control over their circumstances and wellbeing, and can address aspects such as isolation, weight, health or financial worries [19,20].

The Wales School for Social Prescribing Research (WSSPR) is a virtual all-Wales school that is nested within PRIME Centre Wales. WSSPR hosts the Wales Social Prescribing Research Network (WSPRN), a network of over 350 researchers and practitioners in Wales which supports three communities of practice that feed out to members of the public and the social prescribing community across Wales [21]. Through consultation, WSPRN identified the need for a reference tool to help clarify and standardise social prescribing terminology [8,22]. The growth and development of social prescribing over the last decade has been accompanied by the proliferation of a diverse and confusing terminology [8,22,23] that impairs effective communication and creates barriers to engagement. Within Wales, these issues with social prescribing terminology are further compounded by the fact that Wales is a dual-language (Welsh and English) nation. WSSPR, therefore, committed to the development of a glossary of terms for social prescribing [22], for use in Wales and beyond.

Previously, we reported on the first stages of the glossary development process, a scoping review [23] and a study using the consensus-generating methodology of group concept mapping (GCM) [24]. WSSPR and Public Health Wales (PHW) used findings from the scoping review and GCM study, in conjunction with numerous rounds of consultation, to inform the development of a glossary of terms for social prescribing which was published as an interactive PDF [15]. Here we report on the process and considerations for developing the glossary of terms for social prescribing, which includes descriptions for terms, alternative terms for specific aspects of social prescribing, and where appropriate sector-specific preferences for terms. To maximise the usability and accessibility of the glossary a more concise easy-read version [25] was developed in conjunction with Learning Disabilities Wales and a website www.splossary.wales was developed which houses both versions and allows the user to easily switch between Welsh and English.

### The process of developing a glossary of terms for social prescribing

The first stage of the development of the glossary of terms focused on the identification and classification of the terminology associated with social prescribing. This was completed using three methods: A scoping review, a group concept mapping study, and consultation with individuals who worked in or with social prescribing [15,23,24]. Although the methodology and results have previously been reported in detail elsewhere, to provide clarity on the identification and classification of terms associated with social prescribing during the development of the glossary of terms we have included a brief overview in this report.

**Figure 1:**
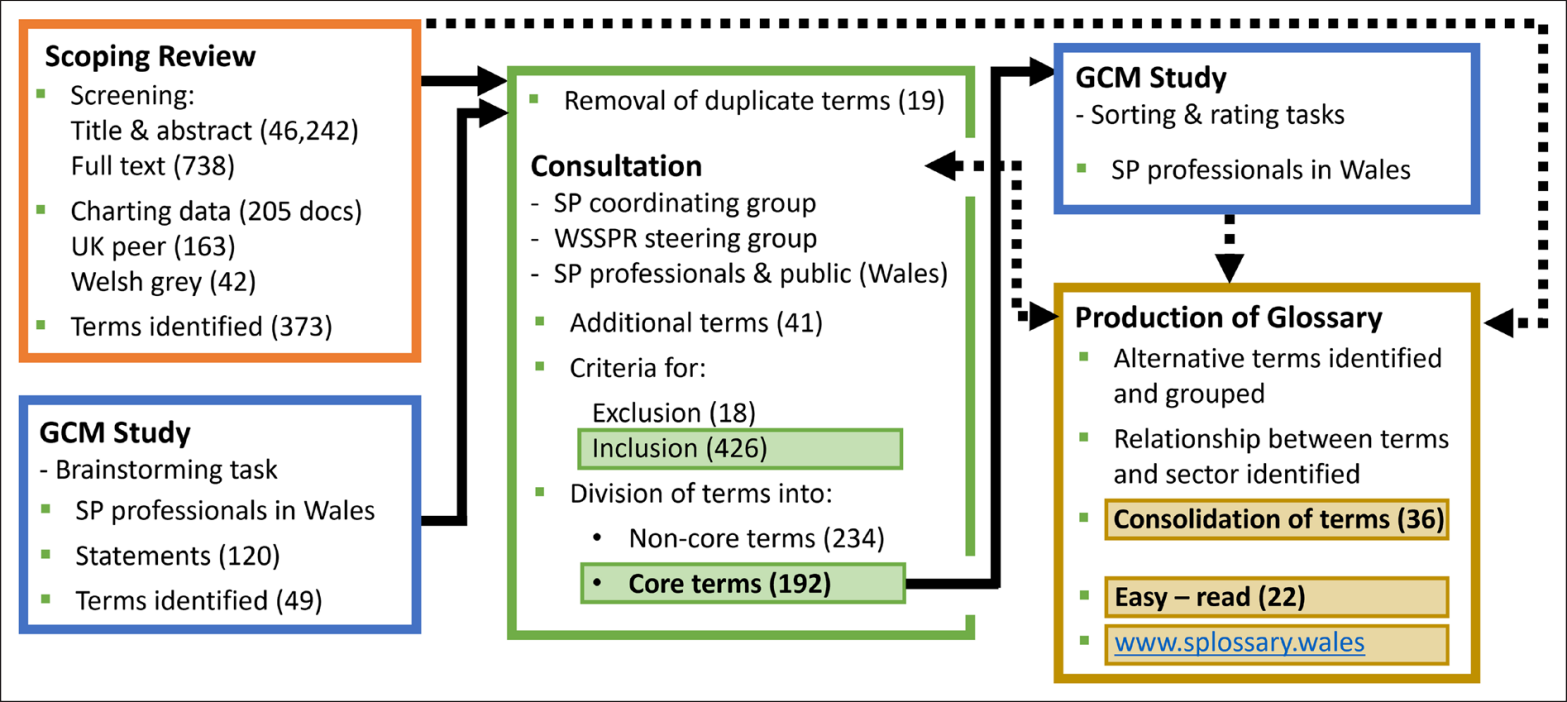
Process flowchart of the development of the glossary of terms.

The second stage used and built on the findings from stage one, to inform the development of the glossary of terms for social prescribing. Both stages are described below and the overall process is depicted in Figure 1.

### Identification and classification of social prescribing terminology

#### Scoping Review

A scoping review [23] was used to map the social prescribing terms and accompanying descriptions terms from 205 documents: UK peer-reviewed journal articles (n = 163) and Welsh grey literature documents (n = 42). The scoping process also incorporated an analytical reinterpretation of the literature, ensuring that the extracted data was presented in a structured way [26,27,28]. Identified terms and accompanying contextual information from the document were charted and classified by the location of the research or intervention described in the article and the perspective that the article was authored from (e.g., healthcare, social care, third sector).

The scoping review identified 373 terms associated with social prescribing (UK peer-reviewed literature: n = 298, Welsh grey literature: n = 113). 70% of all terms (n = 260) occurred solely in peer-reviewed literature, 20% (n = 75) occurred solely in the Welsh grey literature, and there was an overlap of 10% (n = 38) of terms co-occurring in both sources. Only the terms ‘social prescribing’ and ‘link worker’ co-occurred within both the peer-reviewed literature and the Welsh grey literature, and occurred in the literature from all nations of the UK.

Within the peer-reviewed literature, the largest contributions of terms were from articles that described research and/or interventions based within England or from across the UK, and from articles that were authored either solely or partially from the perspectives of health and health and social care. Relatively few terms were found in peer-reviewed articles that described research/interventions in the other nations of the UK, or that were authored from the perspective of the third sector. In contrast, approximately 40% of terms found in the Welsh grey literature occurred in articles authored from the perspective of the third sector.

#### Group Concept Mapping Study

GCM is a mixed-methods consensus-generating approach that uses structured qualitative and quantitative methods to capture and organise the ideas of a group on a topic of interest and create a meaningful visual conceptualisation of the results [29,30]. The GCM study [24] was conducted using professionals (n = 30) who work in or with social prescribing in Wales. Demographic information on their professional role, experience in social prescribing, and their social prescribing knowledge was collected to aid categorisation and interpretation of the data. Three stages of participant engagement, described in the subsections below, were completed online using the groupwisdom™ software [31].

##### Brainstorming task

In the brainstorming task, participants were asked to respond to a prompt and list terms associated with social prescribing. The task provided an opportunity to capture terms from social prescribing professionals in Wales, that may not have been identified in the scoping review. A total of 49 terms were identified and were subsequently combined with the terms identified in the scoping review and submitted for consultation.

##### Sorting task

**Figure 2:**
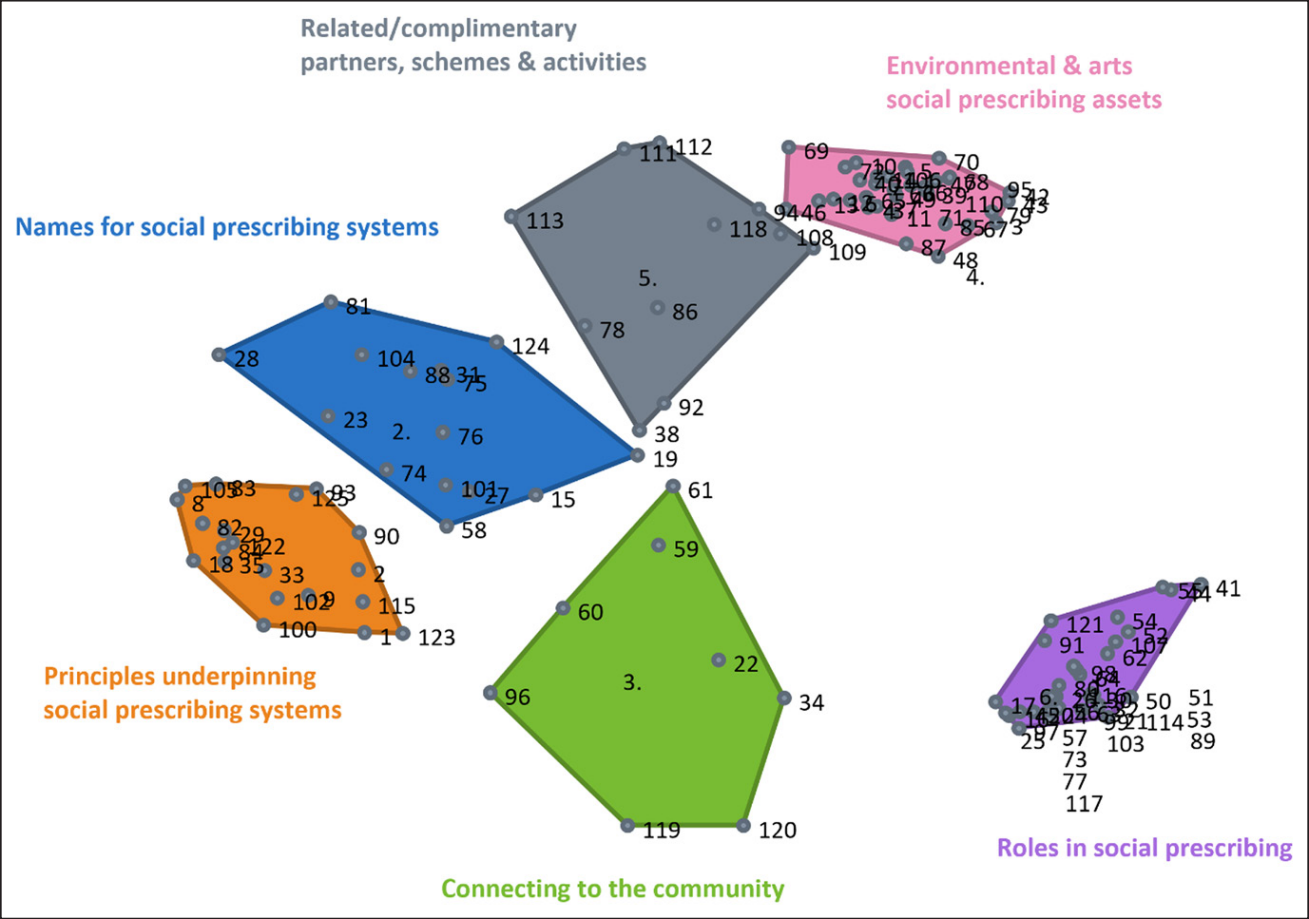
Cluster and point map of core social prescribing terms.

The sorting task used the core terms identified from the scoping review, brainstorming task and consultation (a brief description of the criteria for categorisation as a core term is included in the consultation subsection). Participants were asked to group the terms by “how similar in meaning they are to one another” and give each group a name that described its theme or content. The results provided information on the relationship and the strength of the relationship between terms. The groupwisdom™ software produced a point map, with each point representing one of the core terms. The proximity of the terms to each other indicated the frequency with which they were sorted together. From this data, the software then grouped the points into clusters, with closer clusters indicating a stronger relationship between the terms they contained (Figure 2).

##### Rating task

Participants were asked to rate each of the terms against two, four-point Likert scales, for ‘usefulness of this term in your everyday practice’ and ‘general relevance to social prescribing’. Go-Zone analysis depicted agreement or divergence between the two scales (*r* = .84) and allowed us to identify individual terms by their ratings of usefulness and relevance.

#### Consultation

Consultation was used at various points throughout the process of identifying and categorising the social prescribing terminology. Consultation occurred with the Social Prescribing Coordinating Group established by PHW, networks associated with WSSPR, and the WSSPR Steering Group, which included public and professional involvement (PPI) members. These groups and networks consisted of individuals who worked in and/or with social prescribing in various capacities, including social prescribing practitioners, health and social care professionals and representatives of professional bodies.

The consultation process helped to identify an additional 41 terms that were not captured during the scoping review or the brainstorming element of the GCM study. The Social Prescribing Coordinating Group helped develop the criteria used to define the scope for categorisation of the terms [15] to be included in the glossary:

**Core Social Prescribing Terms Definition:** A term used in everyday language in social prescribing by social prescribing practitioners, professionals and people who engage with social prescribing, that specifically relates to and/or describes an essential part of the social prescribing process.

**Non-Core Social Prescribing Terms Definition:** A term used across health and social care/ statutory/ non-statutory service delivery, which is associated with social prescribing but that does not relate to and/or describe an essential part of the social prescribing process.

### Informed development of the glossary of terms

The development of the glossary of terms focused only on the core social prescribing terms. Even so, a list of 192 terms was too cumbersome to be useful. Identification and classification of social prescribing terms indicated that many related to, or were used to describe, a few specific aspects of the social prescribing process. For example, we identified 28 terms that described the process of social prescribing and 27 terms that describe someone who works in the role of the social prescribing practitioner.

Research by WSSPR [32] indicates that many glossaries are produced by one or several experts in the field and are comprised of a list of 25–50 words. Quite often this process does not involve any external consultation, and the final glossary does not include any accompanying descriptions of the terms. No other glossaries identified in the research included alternative terms of use. This approach may work well for topics where only a single term is used to describe one particular aspect of the subject area and the descriptions of that term are not open to dispute or interpretation. However, the breadth, diversity and ambiguity of the terminology associated with social prescribing necessitated a different approach. The following approach was adopted, with the aim of producing a glossary of terms that not only listed the terms associated with social prescribing but also helped to inform the user and facilitate standardisation of the terminology associated with social prescribing.

Examination of the content of the clusters from the GCM study, and the connection and strength of the connection between terms, allowed us to rudimentarily group terms and identify where multiple terms were associated with the same specific aspects of social prescribing. These groupings were then refined via cross-reference with the data and contextual information gained from the scoping review. Where multiple terms were associated with a particular aspect of social prescribing, the most commonly used and/or most appropriate term for use across sectors was identified. Identification was initially performed using a combination of examination of the rating of terms for usefulness and relevance from the GCM data, followed by cross reference with the scoping review data. Grouping the terms allowed us to produce an initial draft glossary with a consolidated list of 48 glossary terms.

Consultation allowed us to further consolidate the terminology and refine the functionality and content of the glossary. This was an iterative process. The ambiguity of social prescribing terminology necessitated that each term was accompanied by a description. Descriptions were originally obtained from the scoping review data and subsequently refined to ensure that they aligned with the feedback from the consultation of the National Framework for Social Prescribing [33] and, where applicable, Welsh Government policy and legislation. The production of the descriptions for the terms highlighted that some of the 48 glossary terms would be better situated within a description. This process, in addition to several considerations elaborated upon in the discussion, subsequently reduced the list to 36 terms (displayed in Table 1). To facilitate navigation throughout the glossary, each of the 36 terms is displayed within a navigation table and contains a hyperlink to the appropriate place in the glossary.

**Table 1:** The 36 glossary terms of the glossary for social prescribing.


ACTION PLANNING	HOLISTIC

Asset-Based Approach	Nature-Based Interventions

Asset Mapping	Person-Centred Approach

Blue Referral	Practice-Managed Scheme

Books on Referral	Referral

Buddy System	Signposting & Active Signposting

Building the Practice	Social Cafes

Care Navigator	Social Prescribing

Community Assets	Social Prescribing Approaches

Community Support Hubs	Social Prescribing Champion

Community & Voluntary Sector Organisations	Social Prescribing Model

Co-Production	Social Prescribing Outcome Principles

Creative Referral	Social Prescribing Pathway

Digital Social Prescribing	Social Prescribing Practitioner

Education on Referral	Statutory Services

Exercise Referral	Welfare Support Referral

Green Referral	Wellbeing

Health Facilitator	What Matters Conversation


**Figure 3:**
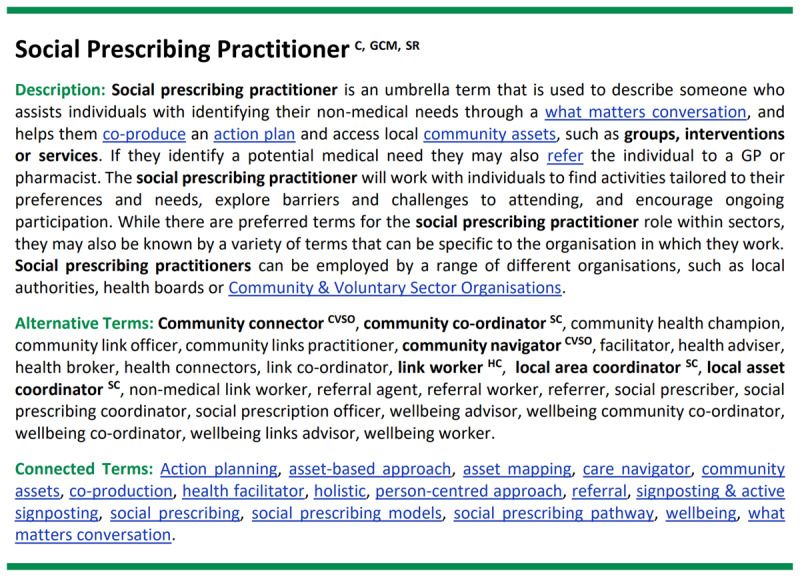
**Glossary entry for the term social prescribing practitioner.** Superscript Key: ^HC^ = healthcare sector, ^SC^ = social care sector, ^CVSO^ = community & voluntary sector organisations.

As many terms were used to describe the same aspect of social prescribing, and to ensure that the glossary was usable and inclusive, we opted to include a list of the ‘Alternative Terms’ below each description. In addition, sector-specific preferences for terms (where appropriate) were established using a combination of scoping review and GCM data and are indicated in the glossary via a super script key (Figure 3). By including terms within the description and an accompanying list of alternative terms, each one of the 192 core terms is represented within the glossary of terms. The glossary document also includes an A–Z list of the 192 core terms, each one of which links to the appropriate place in the glossary.

It was felt that it would be beneficial to indicate the relationship between terms. Each entry in the glossary is accompanied by a list of ‘Connected Terms’, each of which is one of the 36 glossary terms. Users can select a term within the description or from the list of connected terms to navigate to the entry for that term (blue, underlined examples shown in Figure 3).

**Figure 4:**
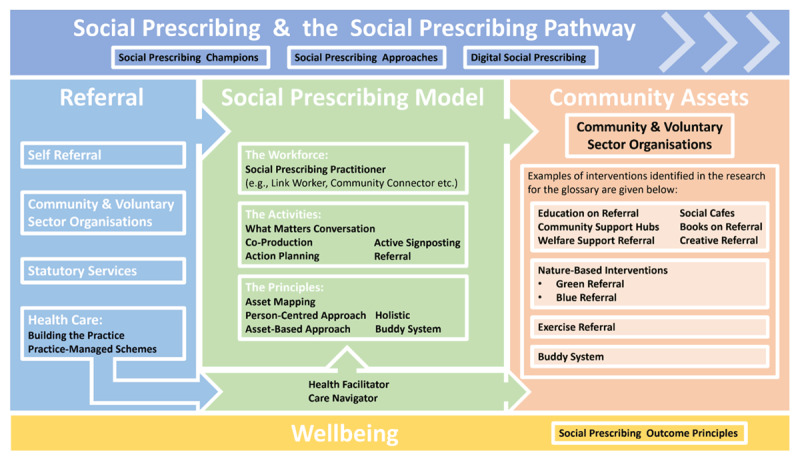
Pathway flowchart of glossary terms used in social prescribing.

An interactive flowchart (Figure 4) was also produced to illustrate how the 36 terms fit within the social prescribing pathway. Again, users can select the terms and easily navigate to the appropriate glossary entry. The user can also easily navigate back to the flowchart or the navigation table by clicking the appropriate links on each page.

The final professional-facing document, published by Public Health Wales and WSSPR [15], includes the following sections:

- A brief background on social prescribing, the need for a glossary of terms and the methodology used to develop the glossary.
- The glossary itself, which includes:
  - Instructions on how to navigate the glossary of terms.
  - A description of the categorisation of terms as core or non-core social prescribing terms.
  - The navigation table.
  - The pathway diagram.
  - The glossary of 36 terms with accompanying descriptions, alternative terms, sector-specific preferences for terms (where appropriate) and connected terms.
  - A–Z lists of the core and non-core social prescribing terms.

The glossary was translated into Welsh by PHW and submitted for consultation with Welsh-speaking members of the public and Welsh-speaking social prescribing academics. Terms and suggestions from the consultation were then submitted to Canolfan Bedwyr University, who specialise in standardising Welsh-language terminology, to make the final decision on which Welsh terms should be used in the glossary.

The glossary of terms was intended as a professional-facing document and the language used within the description is reflective of this. However, it was acknowledged that not all of the terms in the glossary would be relevant to members of the public accessing social prescribing and that the language within the glossary may present a barrier for some of the public. To increase the accessibility of the glossary, WSSPR secured funding to develop a more concise easy-read version [25] and a dedicated website (www.splossary.wales) that houses both versions. The easy-read version of the glossary of terms, developed in conjunction with Learning Disabilities Wales, is an interactive PDF that contains the 22 terms that are most likely to be encountered by those accessing social prescribing. It uses more accessible language in the descriptions for the terms and highlights the most commonly used alternative terms. The website allows users to easily switch between both versions, switch between languages (Welsh/English), indicate their preferred term of use, and provide feedback on the content and usability of the website. To ensure that the website was accessible to both professionals and the public, online forums were held to assess its visual appeal and usability.

## Discussion

The process of developing the glossary was a collaborative effort between WSSPR and PHW, the production of which began as an evidence-based piece of work and evolved into an evidence-based -in-practice piece of work that incorporated numerous rounds of consultation, as well as feedback from the Welsh Government consultation on the National Framework for Social Prescribing [33]. Research by WSSPR indicates that a glossary of terms is usually comprised of a list of 25–50 words which have often been compiled by one or several experts in their field without external consultation. Many have no accompanying description and there was no evidence of the inclusion of alternative terms [32]. The complexities of the language associated with social prescribing necessitated a different approach.

The glossary of terms for social prescribing [15] is an innovative and interactive document that allows the user to easily navigate to new terms. It consists of a list of 36 glossary terms accompanied by descriptions and (where appropriate) lists alternative terms, highlights sector-specific preferences for terms, and demonstrates how terms are connected. Using this format, we were able to include all 192 core terms identified during the research. It provides an informative reference tool for those who work in/with social prescribing and the general public, that will help to standardise that social prescribing terminology. The glossary of terms has already been used to inform the development of the Welsh Government National Framework for Social Prescribing [34], and international counterparts have also expressed interest in the glossary.

The glossary was primarily produced as a professional-facing document aimed at those working in and/or with social prescribing in Wales, but the inclusion of lists of alternative terms makes it applicable for use throughout the UK and beyond. Social prescribing in Wales uses a cross-sectional model of social prescribing that is integrated with existing community and statutory services [8,9]. Consequently, there were several considerations to ensure that the content is both culturally relevant and reflective of Welsh social prescribing policy, legislation and practice:

- The consultation on the National Framework for Social Prescribing [33] identified that the terms ‘prescribing’ and ‘prescription’ were broadly disliked by those working in/with social prescribing in Wales. These terms imply a directive and authoritative approach rather than the co-productive and supportive approach of the Welsh social prescribing model In addition, consultation for the Welsh translation of the glossary identified that ‘referral’ was more commonly used in Welsh for English ‘prescribing’ terms. For example, ‘green prescribing’ translates to ‘rhagnodi gwyrdd’ but within Welsh-speaking communities the term ‘atgyfeiriad gwyrdd’ which translates to ‘green referral’ is more commonly used. In some instances, such as ‘exercise on referral’, the term already existed in English literature and practice. In others, we opted to modify a term to help unite the social prescribing language within Wales. For example, ‘green prescribing’ became ‘green referral’. While reluctant to introduce new terminology when there was already a plethora of terms used in social prescribing, it was felt that a consistent approach was required and that ‘X (on) referral’ was more representative of the practice of social prescribing in Wales, in both languages. It was hoped that this approach would increase acceptance and use of the glossary terms by the social prescribing workforce and therefore help to standardise social prescribing terminology.
- ‘Creative referral’ was an existing umbrella term used to describe the referral of individuals to a range of activities that utilise various forms of creative engagement, such as art on referral, museums on referral and dance on referral. However, consultation on the glossary highlighted that the research had not identified an umbrella term that encapsulated aspects of social prescribing such as debt support and social welfare, legal and financial advice. We, therefore, coined the umbrella term of ‘welfare support referral’.
- The glossary term for ‘social prescribing’ was the subject of much discussion. Our research [15,23,24] identified 27 alternative terms for social prescribing, and that the preferred terms within the third sector were ‘community connection’ and ‘community navigation’. While the glossary term needed to be representative of the social prescribing workforce within Wales, the practice of social prescribing extends beyond the confines of Wales and ‘social prescribing’ is an internationally recognised term. Additionally, ‘social prescribing’ was one of only two terms that occurred in the literature of all four nations of the UK [23], and its inclusion within the glossary ensured alignment with the National Framework for Social Prescribing [34] that was in development at the time. However, considerations were made within the glossary entry for ‘social prescribing’ to acknowledge the use of alternative terms in practice: The accompanying description included the text ‘social prescribing is an umbrella term that describes a person-centred approach to connecting people to local community assets’, and sector-specific preferences for terms were indicated within the list of alternative terms.
- Our research [15,23,24] identified 26 alternatives for the term ‘social prescribing practitioner’. The term ‘link worker’ was the most universally recognised and one of only two terms that occurred in the literature of all four nations of the UK [23]. However, ‘community connector’ and ‘community navigator’ were the most highly rated terms by social prescribing professionals within the third sector [24] and would, therefore, better represent the social prescribing workforce within Wales. Adopting ‘link worker’, which sits very much within the healthcare sector [23,35,36,37], as the glossary term risked ignoring the prevalence and importance of terms used by the social prescribing community in Wales. Additionally, promoting a term such as ‘community connector’ risked alienating potential users outside of the third sector. Consequently, we opted for ‘social prescribing practitioner’ as ‘an umbrella term that is used to describe someone who assists individuals with identifying their non-medical needs through a what matters conversation, and who helps them co-produce an action plan and access local community assets’, while also acknowledging sector-specific preferences within the list of alternative terms.

## Limitations

The glossary of terms should be considered a first step towards the collation and standardisation of social prescribing terminology. The breadth and diversity of the terminology associated with social prescribing and the lack of standardisation of the terminology, even within specific sectors, presented challenges to the production of a cohesive glossary of terms. We, therefore, acknowledge the limitations of the glossary, the process of development and the underpinning research.

The glossary of terms [15] is a professional-facing document and consequently, the language used within it is aimed at professionals working in and/or with social prescribing. Different language was needed to make the document more accessible to the wider public. To this end, WSSPR secured funding to develop a more concise easy-read version of the glossary [25] and a website (www.splossary.wales) that allows users to easily switch between the two versions, as well as between Welsh and English.

Our research indicated that many of the terms identified are seemingly used interchangeably with little standardisation across and within sectors. The development of the glossary was constrained by the data we obtained, making the subcategorisation of terms used to describe the process of social prescribing and the social prescribing practitioner-type roles difficult. Looking forward, refinement and updates to the glossary of terms will inevitably be required, as the language and terminology used within social prescribing evolves. Future refinement will include opportunities for the workforce who encounter and support social prescribing to help clarify descriptions of terms, sector-specific preferences for terms and/or the subcategorisation of terms. The website www.splossary.wales has been designed to facilitate this process, by allowing registered users to indicate their preferred term and provide feedback on the content. incorporates a feedback form that will facilitate this process.

The glossary [15] contains 36 terms which, while the average for a glossary of terms [32], may be considered a little cumbersome for the practice of social prescribing that is in its essence relatively simple. It is unlikely that all terms will be immediately relevant for all users. For example, members of the general public may gravitate towards specific terms they have encountered, such as the descriptions for social prescribing and a what matters conversation, whereas commissioners may utilise terms such as ‘building the practice’ or ‘social prescribing outcome principles’. However, as a tool to facilitate the standardisation of the terminology associated with social prescribing, it was important that the glossary covered the terminology relating to all aspects of social prescribing, and went beyond the immediate pathway by which it is usually defined. Consequently, the glossary of terms can be used as a reference tool by the social prescribing workforce, allied professionals, policymakers and commissioners for training, job descriptions, research, and public information, as well as by anyone to simply clarify their understanding of social prescribing terminology. The more concise easy-read version [25] is better suited to those who are accessing social prescribing, or who feel that they don’t require an in-depth description of the terms.

Of particular relevance to an international audience, to prevent confusion or ambiguity, caution is required when attempting to directly translate and/or reconcile terms from different languages. It is important that engagement takes place with representatives of all levels, from members of the public to policymakers, to ensure that the most appropriate terms from the different languages are used. Judgement will have to be used that considers the intended audience of the glossary. For example, there are two direct Welsh translations of social prescribing: ‘rhagnodi cymdeithasol’ and ‘presgripsiynu cymdeithasol’. Rhagnodi cymdeithasol is the formal translation for social prescribing but is not commonly used in practice. Translators and previously published Welsh policy and legislation have used the term ‘presgripsiynu cymdeithasol’, but the term ‘atgyfeiriad cymdeithasol’ which translates as ‘social referral’ is more commonly used within Welsh-speaking communities. The glossary of terms is a professional-facing document and the decision was, therefore, made to promote the term ‘presgripsiynu cymdeithasol’, ensuring alignment with existing policy and legislation, while also acknowledging the other translations in the list of alternative terms.

A related consideration is the semantic assumptions of terms (e.g. what do people think they mean when they use the term ‘social prescribing’?) compared with the pragmatic use of the term in practice, such as were examined in an international Delphi study of social prescribing terms [38]. Formal definitions and use may differ, from the semantic understanding of those accessing social prescribing. This was an element that was not specifically built into our research. Consequently, further research is required to fully ascertain the semantic understanding of social prescribing terms of different populations in Wales. Additionally, there is the potential for terminology to be subjected to ‘meaning shift’ over time, as exemplified by terminology used by the public concerning mental health and illness [39]. Future iterations of the glossary of terms will provide opportunities to examine both these aspects. To this end, the website www.splossary.wales was developed as a tool to facilitate the collection of data to inform future iterations of the glossary. Users can indicate their preferred terms of use and provide feedback on content. As users are asked to register, responses can be cross-referenced with their demographic information (e.g., public/professional, location, the sector in which they work).

## Conclusion

Social prescribing provides a tool for integrative care that allows individuals to take an active role in improving their health and wellbeing. Unfortunately, the diverse and confusing terminology that accompanies it, impairs cross-sectoral communication and creates barriers to engagement. The glossary of terms for social prescribing takes an important first step in addressing these issues and helping to standardise the language associated with social prescribing across services and professions. To the best of our knowledge, this is the first attempt in the world to produce a comprehensive evidence-based glossary of terms for social prescribing. It provides a foundation from which future work can develop a glossary of terms that can be used internationally to help ensure equity of access to social prescribing across different nations, and to facilitate collaborative research of social prescribing between different nations to better understand the barriers and facilitators of social prescribing.

While the glossary of terms has been developed for social prescribing in Wales, the inclusion of alternative terms makes it a useful tool for other nations of the UK and internationally. The development of a glossary in a bilingual nation (Wales), where the translation of the terminology is markedly divergent between the two spoken languages, highlights the practical issues for glossary development in international settings. The applied methodology, insights gained throughout its development, and the manner in which it has been structured provide additional insights for those developing a glossary of terms, for numerous areas associated with integrative care.
